# Evaluation of Second-Line Treatment for Castration-Resistant Prostate Cancer following the Administration of Upfront Androgen Receptor Signaling Inhibitors

**DOI:** 10.1155/2024/9303603

**Published:** 2024-08-05

**Authors:** Kazuro Kikkawa, Masahiro Tamaki, Kouhei Maruno, Tatsuya Hazama, Toshifumi Takahashi, Yuya Yamada, Masakazu Nakashima, Noriyuki Ito

**Affiliations:** Department of Urology Japanese Red Cross Wakayama Medical Center, 4-20 Komatsubaradori, Wakayama 640-8558, Japan

## Abstract

This study evaluated the effects of docetaxel and androgen receptor signaling inhibitors as second-line treatments in patients with castration-resistant prostate cancer after androgen receptor signaling inhibitors as first-line treatment. This study retrospectively evaluated the clinical outcomes of second-line treatment with docetaxel or androgen receptor signaling inhibitor in patients with castration-resistant prostate cancer who received first-line treatment with androgen receptor signaling inhibitors. Clinical backgrounds and outcomes were compared between docetaxel and androgen receptor signaling inhibitors as second-line treatment. Of 59 patients, 21 (35.6%) and 38 (64.4%) received docetaxel and androgen receptor signaling inhibitors as second-line treatment after first-line treatment with androgen receptor signaling inhibitors, respectively. In the second-line setting, the median progression-free survival was longer with androgen receptor signaling inhibitor than with docetaxel (17 versus 6 months, *P*=0.014). In the first-line setting, the median progression-free survival was longer with androgen receptor signaling inhibitors than with docetaxel (32 versus 25 months, *P*=0.014); however, no significant difference was found in the overall survival. Multivariate analysis revealed that there was no significant association between second-line treatment and survival, and first-line treatment with abiraterone was identified as a prognostic factor for progression-free survival. Subgroup analysis showed that the abiraterone–enzalutamide sequence was more effective than the other three sequences for progression-free survival and overall survival. This study suggests that second-line treatment with an androgen receptor signaling inhibitor for castration-resistant prostate cancer after androgen receptor signaling inhibitors as first-line treatment may be more beneficial, particularly with abiraterone as the upfront treatment.

## 1. Introduction

Prostate cancer (PC) is the second most common malignant tumor in men worldwide, and in 2020, the incidence of PC in men exceeded that of lung cancer in Japan [1]. Androgen deprivation therapy (ADT) is the standard treatment modality for advanced PC. However, most PCs develop resistance to initial hormonal therapy after several years of ADT and often progress to castration-resistant prostate cancer (CRPC) [2].

Docetaxel (DTX) was used from 2004 to improve the prognosis of CRPC patients [3], and it has become the standard treatment for CRPC. Recently, effective systemic agents for CRPC have increased, including androgen receptor signaling inhibitors (ARSIs) such as abiraterone (ABI) and enzalutamide (ENZA). ABI and ENZA as second-line agents following DTX therapy and as first-line agents before DTX therapy were found to improve survival compared with placebo in metastatic CRPC (mCRPC) [4–7]. These agents are thought to be less toxic than DTX [8]. Therefore, since ABI and ENZA were approved for CRPC by the Pharmaceuticals and Medical Devices Agency in Japan in 2014, they have been widely used as standard first-line therapies for CRPC in Japan [9]. However, no reliable guidelines have been established to select the optimal second-line treatment agent for CRPC patients that progressed after first-line treatment with ARSIs. Few retrospective reports have directly compared the efficacy of ARSIs and DTX as the second-line treatment for CRPC [10, 11]. However, the optimal therapeutic sequence of these several available modalities in first- and second-line treatment for CRPC is still debated. Furthermore, there may be a common antitumor mechanism and cross-resistance among ABI, ENZA, and DTX with each other [12–14].

Therefore, the purpose of this study was to evaluate the efficacy of DTX compared with ARSI in the second-line treatment of CRPC patients who received ARSI as first-line treatment. Furthermore, we evaluated whether patients who have disease progression during first-line ARSI should be subsequently treated with another ARSI or DTX.

## 2. Materials and Methods

### 2.1. Study Population

This study retrospectively analyzed patients with CRPC who received first-line treatment with ABI or ENZA between 2012 and 2022. Patients who received ARSIs or DTX before ARSIs for hormone-sensitive PC were excluded. Patient characteristics, including age, prostate-specific antigen (PSA) level, Gleason score, radiological data, and treatment modality for hormone-sensitive PC, were obtained from the electronic medical records. Disease progression was defined as an increase in the PSA level of >25% or an absolute increase of ≥2 ng/mL above the nadir. CRPC was defined as the presence of PSA progression despite continuous treatment with luteinizing hormone-releasing hormone agonist/antagonist. Patients with no evidence of metastatic cancer at or before CRPC were considered to have non-mCRPC (nmCRPC), and patients with any evidence of metastatic cancer at or before CRPC were considered to have mCRPC. DTX was administered at the dose of 70 mg/m^2^ every 3 weeks as a 1 h infusion. Patients received ABI 1000 mg orally once daily plus prednisone 5 mg orally twice daily. They received ENZA 160 mg orally once daily. Dose adjustments and treatment intervals were individualized based on the patient's condition and adverse events (AE), as determined by the physician.

This study was conducted with the approval of the Japanese Red Cross Wakayama Medical Center Institutional Review Board (approval no. 1201) and in accordance with the Declaration of Helsinki.

### 2.2. Outcome Measures

Progression-free survival (PFS) in second-line treatment was assessed by the time from the start of second-line treatment to disease progression. Combined PFS was calculated from the start of first-line treatment with ARSIs to the time of disease progression on the second-line treatment. In addition, overall survival (OS) was defined as the time from the start of primary ARSI treatment to death from any cause, and PSA kinetics for each treatment line and drug were measured using the PSA value at the start of each line, nadir, and at each progression. Treatment-related adverse events (AEs) were evaluated by the Common Terminology Criteria Adverse Events, version 5.

### 2.3. Statistical Analysis

Continuous variables are reported as median and interquartile range. The characteristics of the patients in the two treatment groups were compared by the Mann–Whitney *U* test for continuous variables and the chi-square test and Fisher's exact test for categorical variables. PFS and OS were estimated by the Kaplan–Meier method, and the log-rank test was used to compare survival in the categorized cohorts. Univariate and multivariate analyses were performed using univariate and multivariate Cox proportional hazards analyses. All statistical analyses were performed using JMP Pro 16 (SAS Institute Inc., Cary, NC, USA). *P* values of <0.05 were considered significant.

## 3. Results

### 3.1. Clinical Characteristics

A total of 59 patients were included. All patients received first-line treatment with ABI or ENZA for CRPC. Of these patients, 21 received second-line chemotherapy with DTX, and 38 received second-line treatment with ARSIs. Patient characteristics are shown in Table 1. In patients with CRPC, the median PSA level in the ARSI–ARSI group was higher than that in the ARSI–DTX group (*P*=0.033), and the number of patients who received first-line treatment with ABI was higher in the ARSI–ARSI group than in the ARSI–DTX group. However, no difference in age, Gleason score, rate of metastatic disease, duration of first-line treatment, or PSA level at the initiation of second-line treatment was found between the two groups.

### 3.2. PSA Response

**Figure 1:**
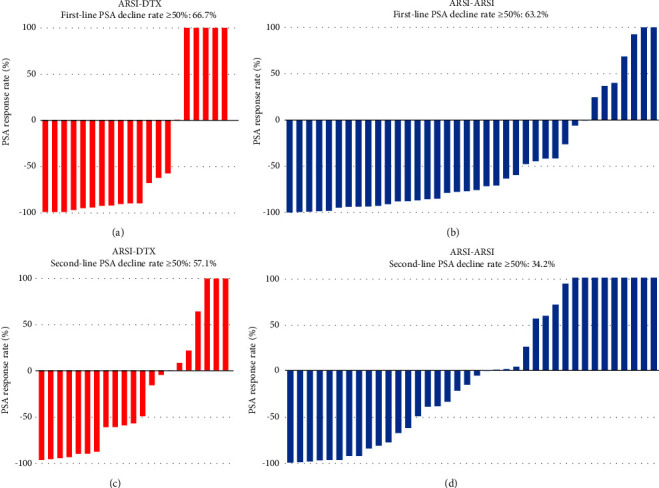
Waterfall plot of the best PSA response rate of each treatment group. (a) ARSI as the first-line treatment in the ARSI–DTX sequence. (b) ARSI as the first-line treatment in the ARSI–ARSI sequence. (c) DTX as the second-line treatment in the ARSI–DTX sequence. (d) ARSI as the second-line treatment in the ARSI–ARSI sequence.

PSA response rates were evaluated in the first-line and second-line treatment settings in the two groups (Figure 1). There was no significantly different in the PSA decline rates of ≥50% in the first-line setting between the ARSI–DTX (14/21, 67%, Figure 1(a)) and ARSI–ARSI (24/38, 63%, Figure 1(b)) groups (*P*=0.788). Although the PSA decline of ≥50% in the second-line treatment was observed more often in the ARSI–DTX group (12/21, 57%, Figure 1(c)) than in the ARSI–ARSI group (13/38, 34%, Figure 1(d)), there was no significant difference between the two groups (*P*=0.088).

### 3.3. Clinical Outcomes

**Figure 2:**
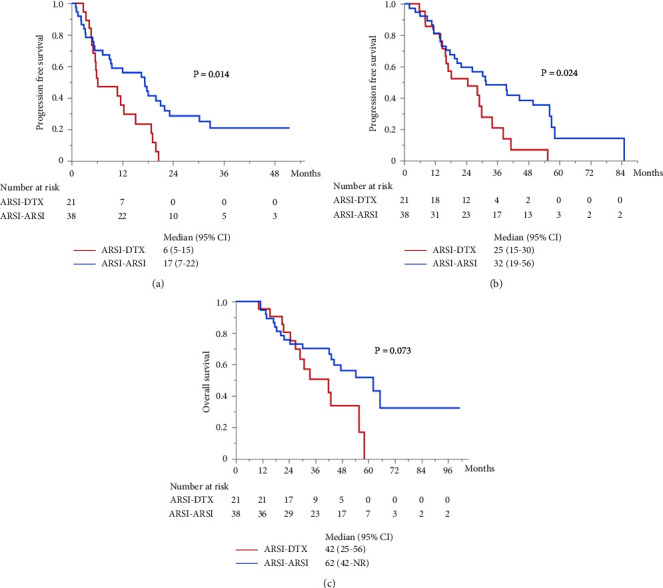
Kaplan–Meier survival curves for patients with CRPC receiving ARSI and DTX as the second-line treatment. (a) PFS in the second-line treatment. (b) Combined PFS. (c) OS from the first-line treatment.

The median PFS in the second-line treatment with ARSIs was 17 months, which was significantly longer than that with DTX (*P*=0.014; Figure 2(a)). The median combined PFS in the ARSI–ARSI group (32 months) was significantly longer than that in the ARSI–DTX group (25 months) (*P*=0.024; Figure 2(b)). Furthermore, the median OS in the ARSI–ARSI group (62 months) was longer than that in the ARSI–DTX group (42 months). However, the difference in OS was not statistically significant (*P*=0.073; Figure 2(c)).

In order to adjust for baseline clinical and demographic factors, variables correlating with PFS and OS were evaluated by Cox proportional hazards analyses after including Gleason score, time to CRPC, metastasis, PSA level, PSA response, treatment sequence, and duration of treatment. In the univariate analysis, time to CRPC of ≥24 months (hazard ratio [HR] 0.49, 95% confidence interval [CI] 0.27–0.90, *P*=0.021), first-line treatment with ENZA (HR 1.87, 95% CI 1.34–3.39, *P*=0.038), and second-line treatment with ARSI (HR 0.45, 95% CI 0.24–0.86, *P*=0.016) were significantly correlated with PFS in the second-line treatment. In the multivariate analysis including these significant prognostic parameters in univariate analysis, time to CRPC of ≥24 months (HR 0.44, 95% CI 0.24–0.83, *P*=0.012) and ENZA as the first-line agent (HR 1.95, 95% CI 1.01–3.75, *P*=0.045) significantly correlated with the PFS in the second-line treatment (Table 2). The same results were observed for combined PFS (Table 3). Time to CRPC of ≥24 months (HR 0.50, 95% CI 0.28–0.91; *P*=0.024) and ENZA as the first-line agent (HR 2.25, 95% CI 1.20–4.20; *P*=0.011) significantly correlated with the combined PFS in the univariate analysis, and time to CRPC of ≥24 months (HR 0.49, 95% CI 0.27–0.92; *P*=0.026) and ENZA as the first-line agent (HR 2.16, 95% CI 1.08–4.32; *P*=0.030) were also significantly correlated in the multivariate analysis (Table 3). Although the second-line treatment with ARSIs was significantly correlated with combined PFS in the univariate analysis (HR 0.48, 95% CI 0.26–0.92; *P*=0.027), the second-line treatment did not correlate with the combined PFS in the multivariate analysis (Table 3). ENZA as the first-line agent (HR 2.35, 95% CI 1.12–4.92; *P*=0.024) and PSA response of ≥50% in the second-line treatment (HR 0.42, 95% CI 0.19–0.93; *P*=0.031) were significantly correlated with OS in the univariate analysis (Table 4). However, no significant differences in OS were found in the multivariate analysis, and the second-line treatment did not correlate with OS.

**Figure 3:**
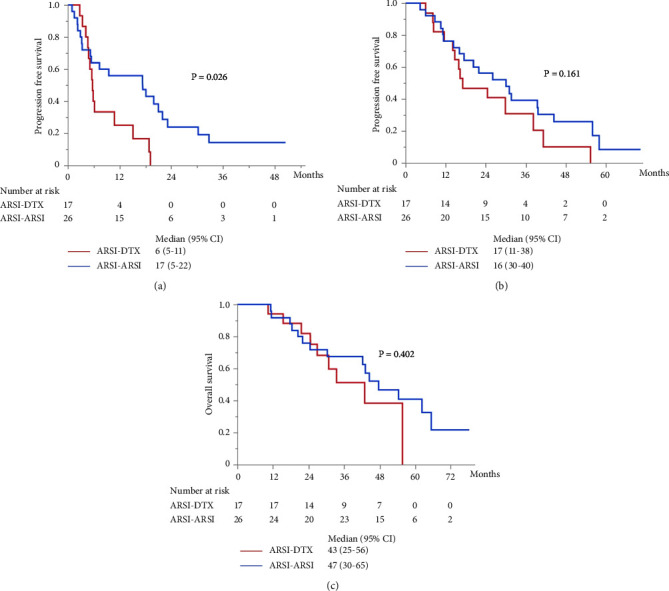
Kaplan–Meier survival curves for patients with mCRPC receiving ARSI and DTX as the second-line treatment in the subgroup analysis. (a) PFS in the second-line treatment. (b) Combined PFS. (c) OS from the first-line treatment.

Although metastasis did not correlate with OS and PFS in the univariate analysis (Tables 2, 3, and 4), subgroup analyses were performed for PFS and OS in patients with mCRPC. In patients with mCRPC, the median PSA level at start of first-line treatment in the ARSI–ARSI group was higher than that in the ARSI–DTX group (14.8 ng/ml vs. 4.8 ng/ml; *P*=0.009). However, no difference in other factors was found between the two groups. The median PFS of mCRPC in the second-line treatment with ARSIs was 17 months, which was significantly longer than that with DTX (*P*=0.026; Figure 3(a)), but there were no significant differences in OS and combined PFS (*P*=0.402 and *P*=0.161; Figures 3(b) and 3(c), respectively). Whereas in patients with nmCRPC, there were no significant differences in OS, PFS, and combined PFS by treatment sequences due to smaller sample size.

**Figure 4:**
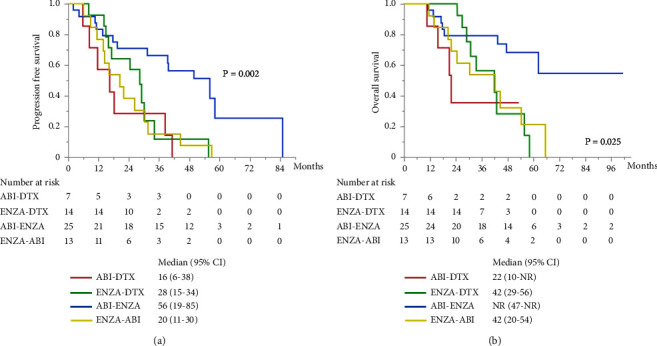
Kaplan–Meier survival curves for patients with CRPC by four treatment sequences in the subgroup analysis. (a) Combined PFS. (b) OS from the first-line treatment.

The other subgroup analyses were performed for combined PFS and OS by first- and second-line treatments. There was significant difference in combined PFS between the groups of each treatment (Figure 4(a)). Combined PFS was significantly better in the ABI–ENZA group than in the ABI–DTX (HR 0.22, 95% CI 0.08–0.57; *P*=0.002), ENZA–DTX (HR 0.34, 95% CI 0.14–0.79; *P*=0.013), and ENZA–ABI (HR 0.28, 95% CI 0.12–0.62; *P*=0.002) groups. Moreover, no difference in combined PFS was found among the three treatment groups, except for the ABI–ENZA group. There was significantly different in OS between the treatment groups (Figure 4(b)), and OS was significantly better in the ABI–ENZA group than in the ABI–DTX (HR 0.24, 95% CI 0.07–0.83; *P*=0.024), ENZA–DTX (HR 0.33, 95% CI 0.12–0.89; *P*=0.028), or ENZA–ABI (HR 0.29, 95% CI 0.12–0.76; *P*=0.012) group. There was no difference in OS between the other three groups, except for the ABI–ENZA group.

AEs were observed in 4 (8.9%) of all 45 patients treated with ABI and included edema, hypertension, fatigue, and elevation of liver enzymes. Three of these patients required a dose reduction for ABI due to AE. Whereas, AEs were observed in 8 (15.4%) of all 52 patients treated with ENZA, including fatigue (*n* = 4), nausea (*n* = 2) dysgeusia (*n* = 1), and hypertension (*n* = 1). Five of these patients required a dose reduction for ENZA due to AE. All of these AEs were grade 1 or 2, and no grade 3 or above AEs were observed. No patient required discontinuation of ABI or ENZA due to AE. In 21 patients treated with DTX, 16 patients (71.4%) had neutropenia, including two patients with grade 3 and 14 patients with grade 4. Although two of these 14 patients with grade 4 neutropenia required dose reduction for DTX, all patients were able to continue treatment with DTX by using granulocyte colony-stimulating factor.

## 4. Discussion

Although ARSIs, including ABI and ENZA, improve prognosis in patients with CRPC [4–7], the optimal treatment sequence for CRPC has not yet been established. This retrospective study evaluated the treatment effects of ARSIs or DTX on CRPC after first-line treatment with ARSIs, and showed that ARSIs as the second-line treatment were associated with a longer PFS than DTX. However, this longer PFS did not affect OS, and there was no significant difference in the multivariate analysis. The results also showed that the time to CRPC and the first-line treatment with ABI were significant prognostic factors for PFS.

To the best of our knowledge, no prospective studies have compared DTX with ARSIs as the second-line treatment for CRPC. Some retrospective studies have reported the efficacy of DTX after ARSI treatment in mCRPC patients. Miyake et al. suggested that the PSA response, PFS, and OS in second-line treatment with DTX were significantly better than those with ARSIs in patients with mCRPC [15]. Similarly, Matsubara et al. reported a significantly better PFS in patients with mCRPC who received DTX as second-line treatment than in those who received ARSIs [16]. However, in this study, PFS and OS in CRPC patients who received ARSIs as the second-line treatment were better than those in patients who received DTX, and these results are not consistent with previous reports. The discrepancies between the previous reports and our study may have several possible reasons. Our study included both patients with nmCRPC and mCRPC. However, these two studies included only patients with mCRPC. Furthermore, we had unequal numbers of patients with ABI or ENZ used as the first-line treatment in each of the DTX and ARSI second-line treatment groups. Several reports have compared the efficacy of ABI and ENZ as the first-line agents for CRPC, and these results varied and were unclear. A meta-analysis suggested that better survival was found with ENZA than with ABI in mCRPC patients [17]. However, a network meta-analysis did not find a difference in the survival between ABI and ENZA for mCRPC [18]. In addition, a network meta-analysis of ARSIs for nmCRPC indicated that ABI provided a comparable survival benefit with other ARSI [19].

Two retrospective reports have compared ARSIs and DTX as second-line treatments for nmCRPC or mCRPC after first-line treatment with ARSI [10, 11]. These reports suggested that DTX as second-line treatment is more beneficial than ARSIs in patients who have received ARSIs as first-line treatment. These results also differ from those of our study. In these two reports, progression was evaluated based on not only PSA but also clinical and radiological findings. However, in our study, progression was assessed using PSA alone. These differences may affect the interpretation of the results. The Advanced Prostate Cancer Consensus Conference 2015 recommended that progression should not be assessed on only increasing PSA and that the presence of at least 2 of the 3 criteria, including symptomatic progression, PSA progression, and radiographic progression, should be considered progression [20]. Many patients receiving ARSI therapy are evaluated using PSA alone, additional imaging studies for PSA progression are performed in daily clinical practice. Matsumoto et al. reported that the median time to CRPC was shorter (DTX 16.1 months and ARSI 19.8 months) and the median PSA levels at both first-line (DTX 14.2 ng/mL and ARSI 16.0 ng/mL) and second-line (DTX 39.0 ng/mL and ARSI 24.9 ng/mL) treatments were higher than those in our study [10]. Therefore, more aggressive cancers may be included in the study. Broyelle et al. reported that the median age at first-line treatment was 66.3 years [11], which was younger than our cohort, and more patients received DTX as the second-line treatment (DTX, *n* = 71; ARSI, *n* = 37). These findings from previous reports and our study suggest that DTX as second-line treatment after progression in first-line treatment with ARSI may be potentially beneficial compared to second-line treatment with ARSI in younger CRPC patients or patients with more aggressive CRPC and that second-line treatment with ARSI may be more appropriate for patients in whom DTX cannot be administered or in those with less aggressive CRPC. However, although the PSA level at start of first-line treatment was higher in the ARSI–ARSI group than in the ARSI–DTX group, the PFS in second-line treatment with DTX was significantly shorter in patients with mCRPC in the subgroup analysis of our study. Generally, younger patients with more aggressive CRPC, such as multiple bone and visceral metastases, are commonly treated with DTX as the first-line treatment, and older patients with less aggressive CRPC or lower performance status are likely to receive ARSI as first-line treatment [21]. These findings from previous report and our study suggest that DTX in earlier phase may be appropriate for more aggressive CRPC. Thus, in a real-world setting, patient characteristics may influence the selection of treatment for CRPC.

In this study, although no significant association was found between survival and PSA response or duration of first-line treatment, the PFS in second-line treatment and combined PFS were significantly shorter in patients with ENZA as first-line treatment. These findings suggest that ARSIs at progression may be beneficial when ABI is used first. Although the ABI–ENZA sequence was more effective than the other three sequences in terms of combined PFS and OS in the subgroup analysis, there were no significant differences between the ABI–DTX sequence and the ENZA–ABI or ENZA–DTX sequences. Mezynski et al. reported that the DTX activity in patients with mCRPC progression after first-line treatment with ABI appears lower [14]. These results and our data suggest that there may be cross-resistance between ABI and DTX. Furthermore, several studies have reported that there may be cross-resistance between ABI and ENZA [12, 13]. ENZA retains its clinical activity as a second-line treatment after ABI, whereas ABI does not retain its second-line activity after ENZA. Our findings are also consistent with these reports. Because these reports were retrospective or had a small sample size, their results are inconclusive regarding the optimal order of treatment. In addition, no studies have reported cross-resistance between ENZA and DTX. Therefore, larger prospective studies are needed to establish the optimal treatment sequence considering cross-resistance.

This study has some limitations. First, this study has a retrospective design with a small sample size. Therefore, the different numbers of patients in the ARSI and DTX groups may lead to heterogeneity in baseline characteristics and measurements. In particular, the subgroup analysis of the four groups with different sequences may have been underpowered to detect true differences. Second, this study did not consider subsequent lines of treatment. For example, the ABI–ENZA group may have improved prognosis because of subsequent treatment including DTX, cabazitaxel, and Radium-223. Furthermore, no strictly regulated criteria are available for the selection of second-line agents and the timing of starting second-line treatment because of the retrospective setting. Thus, some factors, including the time of data collection, timing of starting second-line treatment, and selection of second-line agents, could be biased because of the dependence on individual physicians. Finally, treatment-related quality of life (QOL) was not assessed in this study. Because there are no optimal therapeutic sequence criteria for CRPC, symptoms and QOL are important in the choice of treatment as well as anti-tumor effect. Further research is needed to include symptoms, QOL, and other factors. However, these findings reflect daily clinical practice in the real world; thus, our results may be a useful reference. Because patients with CRPC are often older and have comorbidities, not all treatment modalities are available and suitable. Therefore, it should be considered to select treatment individually based on the patient and disease characteristics in situations where appropriate treatment sequence has not been established.

## 5. Conclusion

This retrospective study suggests that second-line treatment with ARSI after ARSI as first-line treatment for CRPC patients is associated with slightly better outcomes compared with DTX in terms of PFS. However, this improvement was not found in the multivariate analysis. Furthermore, our findings suggest that the ABI–ENZA sequence may have superior anticancer efficacy. Large prospective studies are required to confirm these results and decide the appropriate treatment sequence in CRPC patients.

## Figures and Tables

**Table 1:** Patient characteristics at initiation of first- and second-line treatment.

	ARSI–DTX *n* = 21		ARSI–ARSI *n* = 38		*P* value
Gleason score, *n* (%)					0.537
<8	4	(19.1)	11	(28.9)	
≥8	17	(80.9)	27	(71.1)	
Time to CRPC, median months (IQR)	24	(12.3–32.9)	30.8	(13.1–67.0)	0.176
Age at start of first-line treatment, median years (IQR)	77	(73–79)	79	(73–82)	0.311
PSA at start of first-line treatment, median ng/ml (IQR)	4.8	(1.8–11.5)	8.2	(4.4–29.0)	0.033
Metastasis					0.370
nmCRPC, *n* (%)	4	(19.1)	12	(31.6)	
mCRPC, *n* (%)	17	(80.9)	26	(68.4)	
Metastatic site, *n* (%)					
Bone	14	(66.7)	21	(55.3)	0.393
Visceral	1	(4.8)	3	(7.9)	0.647
First-line agent, *n* (%)					0.017
ABI	7	(33.3)	25	(65.8)	
ENZA	14	(66.7)	13	(34.2)	
Duration of first-line treatment, median months (IQR)	10.9	(6.3–23.8)	10.8	(4.9–22.2)	0.975
Age at start of second-line treatment, median years (IQR)	78	(74–81)	80	(75–83)	0.270
PSA at start of second-line treatment, median ng/ml (IQR)	8.8	(1.9–59.5)	7.8	(2.3–29.3)	0.899
Second-line agent, *n* (%)					
ABI	—		13	(34.2)	
ENZA	—		25	(65.8)	
DTX	21	(100)	—		

DTX, docetaxel; ARSI, androgen receptor signaling inhibitor; CRPC, castration-resistant prostate cancer; IQR, interquartile range; PSA, prostate specific antigen; nmCRPC, nonmetastatic castration-resistant prostate cancer; mCRPC, metastatic castration-resistant prostate cancer; ABI, abiraterone; ENZA, enzalutamide.

**Table 2:** Univariate and multivariate analysis for PFS in second-line treatment.

Variables	Univariate		*P* value	Multivariate		*P* value
	HR	95% CI		HR	95% CI	
Gleason score						
<8	1.0					
≥8	1.17	0.61–2.28	0.628			
Time to CRPC						
<24 months	1.0			1.0		
≥24 months	0.49	0.27–0.90	0.021	0.44	0.24–0.83	0.012
Metastasis						
nmCRPC	1.0					
mCRPC	1.61	0.81–3.19	0.167			
First-line agent						
ABI	1.0			1.0		
ENZA	1.87	1.34–3.39	0.038	1.95	1.01–3.75	0.045
PSA response of first-line treatment						
<50%	1.0					
≥50%	1.21	0.65–2.21	0.546			
Duration of first-line treatment						
<12 months	1.0					
≥12 months	0.63	0.34–1.18	0.154			
PSA at start of second treatment						
<8.0 ng/ml	1.0					
≥8.0 ng/ml	1.08	0.60–1.94	0.795			
Second-line treatment						
DTX	1.0			1.0		
ARSI	0.45	0.24–0.86	0.016	0.64	0.32–1.27	0.207

HR, hazard ratio; CI, confidence interval; CRPC, castration-resistant prostate cancer; nmCRPC, non-metastatic castration-resistant prostate cancer; mCRPC, metastatic castration-resistant prostate cancer; ABI, abiraterone; ENZA, enzalutamide; PSA, prostate specific antigen; DTX, docetaxel; ARSI, androgen receptor signaling inhibitor.

**Table 3:** Univariate and multivariate analysis for combined PFS.

Variables	Univariate		*P* value	Multivariate		*P* value
	HR	95% CI		HR	95% CI	
Gleason score						
<8	1.0					
≥8	1.55	0.79–3.04	0.205			
Time to CRPC						
<24 months	1.0			1.0		
≥24 months	0.50	0.28–0.91	0.024	0.49	0.27–0.92	0.026
PSA at CRPC						
<7.0 ng/ml	1.0					
≥7.0 ng/ml	1.02	0.56–1.84	0.952			
Metastasis						
nmCRPC	1.0					
mCRPC	1.76	0.87–3.58	0.117			
First-line agent						
ABI	1.0			1.0		
ENZA	2.25	1.20–4.20	0.011	2.16	1.08–4.32	0.030
PSA response of first-line treatment						
<50%	1.0					
≥50%	0.69	0.38–1.28	0.243			
Second-line treatment						
DTX	1.0			1.0		
ARSI	0.48	0.26–0.92	0.027	0.78	0.38–1.61	0.515
PSA response of second-line treatment						
<50%	1.0					
50%	0.54	0.29–1.03	0.063			

HR, hazard ratio; CI, confidence interval; CRPC, castration-resistant prostate cancer; PSA, prostate specific antigen; nmCRPC, non-metastatic castration-resistant prostate cancer; mCRPC, metastatic castration-resistant prostate cancer; ABI, abiraterone; ENZA, enzalutamide; DTX, docetaxel; ARSI, androgen receptor signaling inhibitor.

**Table 4:** Univariate and multivariate analysis for OS.

Variables	Univariate		*P* value	Multivariate		*P* value
	HR	95% CI		HR	95% CI	
Gleason score						
<8	1.0					
≥8	2.02	0.92–4.43	0.080			
Time to CRPC						
<24 months	1.0					
≥24 months	0.61	0.30–1.23	0.167			
PSA at CRPC						
<7.0 ng/ml	1.0					
≥7.0 ng/ml	1.27	0.62–2.58	0.512			
Metastasis						
nmCRPC	1.0					
mCRPC	1.46	0.63–3.41	0.378			
First-line agent						
ABI	1.0			1.0		
ENZA	2.35	1.12–4.92	0.024	2.00	0.94–4.32	0.071
PSA response of first-line treatment						
<50%	1.0					
≥50%	0.65	0.32–1.33	0.237			
Second-line treatment						
DTX	1.0					
ARSI	0.51	0.24–1.08	0.078			
PSA response of second-line treatment						
<50%	1.0			1.0		
≥50%	0.42	0.19–0.93	0.031	0.49	0.22–1.10	0.085

HR, hazard ratio; CI, confidence interval; CRPC, castration-resistant prostate cancer; PSA, prostate specific antigen; nmCRPC, non-metastatic castration-resistant prostate cancer; mCRPC, metastatic castration-resistant prostate cancer; ABI, abiraterone; ENZA, enzalutamide; DTX, docetaxel; ARSI, androgen receptor signaling inhibitor.

## Data Availability

Derived data supporting the findings of this study are available from the corresponding author on request.

## Abbreviations

ABI: Abiraterone
ADT: Androgen deprivation therapy
AE: Adverse event
ARSI: Androgen receptor signaling inhibitor
CRPC: Castration-resistant prostate cancer
DTX: Docetaxel
ENZA: Enzalutamide
HSPC: Hormone-sensitive prostate cancer
mCRPC: Metastatic castration-resistant prostate cancer
nmCRPC: Nonmetastatic castration-resistant prostate cancer
OS: Overall survival
PC: Prostate cancer
PFS: Progression-free survival
PMDA: Pharmaceuticals and medical devices agency
PSA: Prostate-specific antigen
QOL: Quality of life.
